# A common TMPRSS2 variant has a protective effect against severe COVID-19

**DOI:** 10.1016/j.retram.2022.103333

**Published:** 2022-05

**Authors:** Alessia David, Nicholas Parkinson, Thomas P Peacock, Erola Pairo-Castineira, Tarun Khanna, Aurelie Cobat, Albert Tenesa, Vanessa Sancho-Shimizu, GenOMICC Consortium, ISARIC4C Investigators, Jean-Laurent Casanova, Laurent Abel, Wendy S. Barclay, J.Kenneth Baillie, Michael JE Sternberg

**Affiliations:** a Centre for Integrative System Biology and Bioinformatics, Department of Life Sciences, Imperial College London, London, SW7 2AZ, UK; b Roslin Institute, University of Edinburgh, Easter Bush, Edinburgh, EH25 9RG, UK; c Department of Infectious Diseases, Imperial College London, London, W2 1PG, UK; d St. Giles Laboratory of Human Genetics of Infectious Diseases, Rockefeller Branch, The Rockefeller University, New York, NY 10065, USA; e Laboratory of Human Genetics of Infectious Diseases, Necker Branch, INSERM U1163, Necker Hospital for Sick Children, Paris, EU France; f University of Paris, Imagine Institute, Paris, EU France; g Department of Paediatric Infectious Diseases & Virology, Imperial College London, London, UK; h Centre for Paediatrics and Child Health, Faculty of Medicine, Imperial College London, London, UK; i Howard Hughes Medical Institute, New York, NY, USA; j Intenstive Care Unit, Royal Infirmary of Edinburgh, 54 Little France Drive, Edinburgh, EH16 5SA, UK

**Keywords:** SARS-CoV-2, COVID-19, TMPRSS2, Targeting the host to prevent COVID19 severity

## Abstract

**Background:**

The human protein transmembrane protease serine type 2 (TMPRSS2) plays a key role in SARS-CoV-2 infection, as it is required to activate the virus’ spike protein, facilitating entry into target cells. We hypothesized that naturally-occurring TMPRSS2 human genetic variants affecting the structure and function of the TMPRSS2 protein may modulate the severity of SARS-CoV-2 infection.

**Methods:**

We focused on the only common TMPRSS2 non-synonymous variant predicted to be damaging (rs12329760 C>T, p.V160M), which has a minor allele frequency ranging from 0.14 in Ashkenazi Jewish to 0.38 in East Asians. We analysed the association between the rs12329760 and COVID-19 severity in 2,244 critically ill patients with COVID-19 from 208 UK intensive care units recruited as part of the GenOMICC (Genetics Of Mortality In Critical Care) study. Logistic regression analyses were adjusted for sex, age and deprivation index. For in vitro studies, HEK293 cells were co-transfected with ACE2 and either TMPRSS2 wild type or mutant (TMPRSS2_V160M_). A SARS-CoV-2 pseudovirus entry assay was used to investigate the ability of TMPRSS2_V160M_ to promote viral entry.

**Results:**

We show that the T allele of rs12329760 is associated with a reduced likelihood of developing severe COVID-19 (OR 0.87, 95%CI:0.79–0.97, *p* = 0.01). This association was stronger in homozygous individuals when compared to the general population (OR 0.65, 95%CI:0.50–0.84, *p* = 1.3 × 10^−3^). We demonstrate in vitro that this variant, which causes the amino acid substitution valine to methionine, affects the catalytic activity of TMPRSS2 and is less able to support SARS-CoV-2 spike-mediated entry into cells.

**Conclusion:**

TMPRSS2 rs12329760 is a common variant associated with a significantly decreased risk of severe COVID-19. Further studies are needed to assess the expression of TMPRSS2 across different age groups. Moreover, our results identify TMPRSS2 as a promising drug target, with a potential role for camostat mesilate, a drug approved for the treatment of chronic pancreatitis and postoperative reflux esophagitis, in the treatment of COVID-19. Clinical trials are needed to confirm this.

## Introduction

The severe acute respiratory syndrome like coronavirus (SARS-CoV-2) has infected over 190 million individuals globally and has caused more than 4.2 million deaths as of August 2021 [1]. SARS-CoV-2 infection has a broad clinical spectrum, ranging from asymptomatic or mildly symptomatic, to a life-threatening presentation requiring admission to intensive care. Age, and to a much lesser extent male gender and various underlying clinical conditions, such as cardiovascular disease, obesity and diabetes, are known risk factors associated with an increased COVID-19 morbidity and mortality [2,3]. The role of an individual's genetic background has recently emerged as an additional, yet not clearly understood, risk factor for COVID-19 [4], [5], [6]. Rare genetic variants in genes involved in the regulation of type I interferon (IFN) immunity, including autosomal recessive IRF7 and IFNAR1 deficiencies, have been identified in patients with life-threatening COVID-19 [6]. Autoantibodies to type I IFNs also account for at least 10% of cases of critical COVID-19 pneumonia[7]. Genome-wide association studies (GWAS) have discovered genetic haplotypes spanning several genes that are associated with COVID-19 severity [3,4,8].

The transmembrane protease serine type 2 (TMPRSS2) protein plays a key role in coronavirus infections [9], [10], [11], including SARS-CoV-2, as it is required for priming the virus’ spike (S) glycoprotein through its cleavage, thus facilitating endosome-independent entry into target cells [12,13]. TMPRSS2, which is part of the type 2 transmembrane serine protease (TTSP) family, is characterized by androgen receptor elements located upstream to its transcription site [14]. As well as cleaving and activating viral glycoproteins of coronaviruses and influenza A and B viruses [15], TMPRSS2 is subject to autocleavage, which results in the liberation of its soluble catalytic domain [16]. The conditions under which autocleavage of TMPRSS2 and other members of the TTSPs family occurs are yet to be elucidated.

TMPRSS2 is expressed in lung and bronchial cells [17], but also in the colon, stomach, pancreas, salivary glands and numerous other tissues [18]. Moreover, it is co-expressed in bronchial and lung cells with the angiotensin-converting enzyme 2 (ACE2) [17], which is the best described SARS-CoV-2 cellular receptor [19]. In the olfactory epithelium of mice, the expression of TMPRSS2, but not ACE2, appears to be age-related and greater in older compared to younger animals [20]. Similarly, a recent study showed that expression of TMPRSS2 in mouse and human lung tissue is also age-related [21]. Studies in TMPRSS2 knock out (KO) mice reported reduced SARS-CoV and MERS-CoV replication in the lungs compared to wild-type mice, and a reduced proinflammatory viral response, especially cytokine and chemokine release via the Toll-like receptor 3 pathway [22,23]. We have recently shown that TMPRSS2 expression permits cell surface entry of SARS-CoV-2, allowing the virus to bypass potent endosomal restriction factors [24]. In vitro studies have shown that TMPRSS2 inhibitors prevent primary airway cell and organoid infection by SARS-CoV and SARS-CoV-2 [25,24,26]. In animal studies, mice infected with SARS-CoV and treated with the serine protease inhibitor camostat mesilate had a high survival rate [27]. Recently, camostat mesilate (which, in Japan, is already approved for patients with chronic pancreatitis and postoperative reflux esophagitis) was shown to block SARS-CoV-2 lung cell infection in vitro [12,24]. Furthermore, camostat mesylate and its metabolite GBPA have been shown to block SARS-CoV-2 spread in human lung tissue ex vivo [28]. Several clinical trials using camostate in COVID-19 patients are currently underway [29].

In view of the data from animal models and cell-based studies supporting a protective role of a knock out TMPRSS2 on coronavirus infection (including SARS and MERS), we hypothesized that naturally-occurring TMPRSS2 genetic variants affecting the structure and function of the TMPRSS2 protein may modulate the severity of SARS-CoV-2 infection.

## Methods

### TMPRSS2 three-dimensional structure and variant analysis

The recently released 3D structure of TMPRSS2 (PDB: 7meq) was used to assess the impact missense variants. The Phyre homology modelling algorithm [30] was used to resolve missing amino acid regions in the SRSC domain that were not experimentally solved (described in Supplementary material). The FASTA sequence of TMPRSS2 was obtained from the UniProt protein knowledge database [31] (UniProt Id O15393, corresponding to 492 amino acid transcript Ensembl ID ENST00000332149.10). The recently released AlphaFold model [32] was compared to the Phyre model. The impact of each missense variant on the TMPRSS2 protein structure was assessed by analysing the following 16 features, using our in-house algorithm Missense3D [33]: breakage of a disulfide bond, hydrogen bond or salt bridge, introduction of a buried proline, clash, introduction of hydrophilic residue, introduction of a buried charged residue, charge switch in a buried residue, alteration in secondary structure, replacement of a charged with uncharged buried residue, introduction of a disallowed phi/psi region, replacement of a buried glycine with any other residue, alteration in a cavity, replacement of *cis* proline, buried to exposed residue switch, replacement of a glycine located in a bend. In addition, we used the SIFT [34] and Polyphen2 [35] variant predictors, which mainly use evolutionary conservation to assess the effect of each variant. The effect of variant rs12329760 was further assess using: i. CONDEL [36], which reports a weighted average of the scores from fatHMM and MutationAssessor, and ii. FoldX5 force field [37], which calculates the stability of a protein based on the estimation of its free energy. A ΔΔG> 0.5 kcal/mol (calculated as: ΔΔG= ΔG_wt_ - ΔG_mut_) was predicted to have a destabilizing effect.

### Participants

*Genetics Of Mortality In Critical Care (GenOMICC) and the International Severe Acute Respiratory Infection Consortium (ISARIC) Coronavirus Clinical Characterisation Consortium (4C) (ISARIC 4C)*

Cases: this cohort was established between March 2020 and July 2020 (first COVID-19 wave) and comprises of 2244 critically ill, hospitalized COVID-19 positive patients from 208 UK intensive care units (ICUs): 2109, patients were recruited as part of the GenOMICC project, and an additional 135 cases as part of the International Severe Acute Respiratory Infection Consortium (ISARIC) Coronavirus Clinical Characterisation Consortium (4C) study. The clinical characteristics and comorbidities of these patients have been extensively reported in Pairo-Castineira et al. [8]. Only unrelated individuals (up to 3rd degree, based on kinship analysis (King 2.1)) were included. Samples were excluded if the genotype-based sex inference did not match the reported sex, or if a XXY karyotype was present. Moreover, patients of mixed genetic ancestry, and from ancestry groups with small numbers of cases (such as North American Indian, *n* = 13) defined using admixture supervised mode with 1000 genomes as reference, were excluded.

Controls: ancestry-matched controls (ratio 1 case to 5 controls) without a positive COVID-19 test were obtained from the UK BioBank population study. COVID-19 test results in BioBank are obtained from Public Health England, Public Health Scotland and SAIL for English, Scottish and Welsh data, respectively. The vast majority of results are from nose/throat swabs analysed by PCR. For patients admitted to hospital, results can also be from samples obtained from the lower respiratory tract. Only unrelated individuals (up to 3rd degree) were included. Individuals with sex mismatch were excluded. For validation, 45,875 unrelated individuals of European ancestry from the 100,000 Genomes Project were used as an alternative control group.

DNA extraction, genotyping and quality control have been described in detail previously[8]. Genetic ancestry was inferred using ADMIXTURE and reference individuals from the 1000 Genomes project. Imputation was performed using the TOPMed reference panel.

### Cells, pseudovirus and plasmid

Human embryonic kidney 293T cells (293Ts; ATCC) were maintained in Dulbecco's modified Eagle's medium (DMEM), 10% foetal calf serum (FCS), 1% non-essential amino acids (NEAA), 1% penicillin-streptomycin (P/S). Human Caco-2 (ATCC HTB-37) and Calu-3 (ATCC HTB-55) were maintained in DMEM, 20% FCS, 1% NEAA, 1% P/S. All cell lines were maintained at 37 °C, 5% CO_2_.

Lentiviral pseudotype production was performed as previously described[24][38]. Briefly, pseudovirus was generated by co-transfecting 293Ts with lentiviral packaging constructs pCSFLW (minimal HIV genome with firefly luciferase reporter), pCAGGs-GAGPOL (HIV packing proteins) and the relevant viral glycoprotein in pcDNA3.1 – either the G glycoprotein from Indiana vesiculovirus (VSV-G) or SARS-CoV-2 spike protein. Co-transfections were performed at a plasmid ratio of 1.5:1:1 for pCSFLW:GAGPOL:glycoprotein. Pseudovirus was harvested at 48 and 72 h post-transfection, pooled, filtered, then frozen down. ACE2 FLAG was used as previously described [24]. TMPRSS2 expression plasmid was a kind gift from Roger Reeves (Addgene plasmid #53,887; http://n2t.net/addgene:53887; RRID:Addgene_53,887) [39]. Non-cleavable ACE2-FLAG and TMPRSS2 mutants were generated by overlap extension PCR or site-directed mutagenesis.

### Phenotypic assays

293Ts were co-transfected with FLAG-tagged, non-cleavable ACE2 and TMPRSS2, as previously described [24]. Briefly, confluent 10cm^2^ dishes of 293T cells were co-transfected with 1 µg each of TMPRSS2 and ACE2-FLAG. 24 h later, cells were resuspended in fresh media and either spun down for lysis and western blot or added to 96 well plates along with pseudovirus. 24 h later, media was refreshed and a further 24 h later, cells were lysed with reporter lysis buffer (Promega), and luminescence (measured as relative luminescence units, RLU) was read on a FLUOstar Omega plate reader (BMF Labtech) using the Luciferase Assay System (Promega).

Cell pellets for western blot were lysed in RIPA buffer (150 mM NaCl, 1% NP-40, 0.5% sodium deoxycholate, 0.1% SDS, 50 mM TRIS, pH 7.4) supplemented with an EDTA-free protease inhibitor cocktail tablet (Roche). Cell lysates were combined with 4x Laemmli buffer (Bio-Rad) with 10% β-mercaptoethanol and boiled for 5 min. Membranes were probed with mouse anti-tubulin (abcam; ab7291), rabbit anti-TMPRSS2 (abcam; ab92323) and/or mouse anti-FLAG (F1804, Sigma). Near infra-red (NIR) secondary antibodies, IRDye® 680RD Goat anti-mouse (abcam; ab216776) and IRDye® 800CW Goat anti-rabbit (abcam; ab216773) were subsequently used. Blots were imaged using the Odyssey Imaging System (LI-COR Biosciences). Densitometry was performed using ImageJ.

### Reagent, cell lines and antibody validation

All reagents, cell lines and antibodies used in this study are commercially available and validation data are available on the manufacturers’ websites.

### Statistical analysis

Sample size: Critically ill Covid-19 patients, *n* = 2,244; random controls matched by ancestry from UK Biobank, *n* = 11,220. The sample size was determined pragmatically by the number of cases recruited during the first wave of the outbreak in the UK (as described in [8]). No randomization was performed. Blinding was not used in this study because the exposure (genotype) and outcome (ICU admission) are objective. Confounding was controlled by the use of covariates: age, sex, deprivation score and genetic ancestry [8]. The association between the TMPRSS2 rs12329760 variant and COVID-19 severity was assessed using logistic regression. Genetic associations in the GenOMICC/ISARIC 4C cohort were analysed as previously described[8]. Briefly, logistic regression with additive and recessive models was performed in PLINKv1.9, adjusting for sex, age, mean-centred age-squared, top 10 principal components (principal component analysis [PCA] performed to adjust for population stratification) and deprivation index decile based on UK postcode. Each major ancestry group alternative in the 100,000 Genomes control group was performed with mixed model association tests in SAIGE (v0.39) [40], including age, sex, age-squared, age-sex interaction and the first 20 principal components as covariates. Trans-ethnic meta-analysis of GenOMICC data for different ancestries was performed by METAL using an inverse-variance weighted method and the P-value for heterogeneity was calculated with Cochran's Q-test for heterogeneity implemented in the same software [41].

Additional publicly available genetic data were obtained from the COVID-19 Host Genetics Initiative meta-analyses, release 6 (June 15, 2021) [42]. The COVID-19 Host genetics initiative classifies COVID-19 severity according to the use of invasive and non-invasive ventilation during hospital admission. Here we report the four different phenotype comparisons:

• A2: 8,779 critically ill confirmed cases (inclusion criteria: hospitalized for COVID-19 and either death or on respiratory support including intubation, CPAP, BiPAP, continue external negative pressure, Optiflow/very high flow Positive End Expiratory Pressure Oxygen) versus 1,001,875 population controls,
• B1: 14,480 hospitalised cases versus 73,191 non-hospitalised cases,
• B2 24,274 hospitalised cases versus 2,061,529 population controls, and
• C2: 112,443 COVID-19 cases of unspecified severity versus 2,473,889 population controls.

Analyses used all data with the exclusion of the 23&Me study, for which full data were not publicly available. Meta-analysis in all cases was performed using a fixed effect, inverse variance-weighted model, either as a trans-ethnic meta-analysis or subsetted by ancestry group.

Data are presented as mean ± standard deviation. Log-normality was assessed using the Shapiro-Wilk test and QQ plot. A two-tailed Student's *t*-test was used to compare the means of two groups. One-way ANOVA was used to compare the means of more than two groups.

### Colocalisation analysis

Colocalisation analysis for genetic associations was performed by an Approximate Bayes Factor approach using the package *coloc* version 5, in R 4.1.0 [43]. Summary statistics (beta and variance) were from GWAS data [8] and from lung eQTL data from GTex v8 [44], in individuals of European ancestry. To reduce the likelihood of violation of the single causal variant assumption arising from multiple independent association signals, the analysis was restricted to a region extending to 5 kb upstream and downstream of the TMPRSS2 gene. With the assumption that exactly one measured SNP in the region was causative for each trait, SNP-level priors (p1 and p2) of 1/(n SNPs) were used for the probability of association with each individual trait, with an arbitrary prior of 0.1 x p1 for p12, the SNP-level prior probability of association with both traits. Sensitivity analysis was performed to assess the impact of prior selection, comparing the selected priors to the more stringent default priors (10^−4^ for p1 and p2, 10^−5^ for p12), and varying the p12 range from p1 to p1 x p2.

### Ethics

Research ethics committees (Scotland 15/SS/0110, England, Wales and Northern Ireland: 19/WM/0247). Current and previous versions of the study protocol are available at genomicc.org/protocol. All participants gave informed consent.

### Role of funders

The Wellcome Trust, UKRI, MRC/UKRI, Howard Hughes Medical Institute, Rockefeller University, St. Giles Foundation, Fisher centre for Alzheimer's Research Foundation, Meyer Foundation, Square Foundation, Grandir Fonds de solidarité pour l'enfance, SCOR Corporate Foundation for Science, Institut National de la Santé et de la Recherche Médicale (INSERM), University of Paris, National Institutes of Health, French Foundation for Medical Research, FRM and French National Research Agency (ANR) GENCOVID, Agence Nationale de la Recherche, Health Data Research UK and BBSRC provided funding to support the salaries of the authors but had no role in the design, data collection, analysis, interpretation of the results or writing of the report. The content of this publication is solely the responsibility of the authors.

## Results

**Fig. 1:**
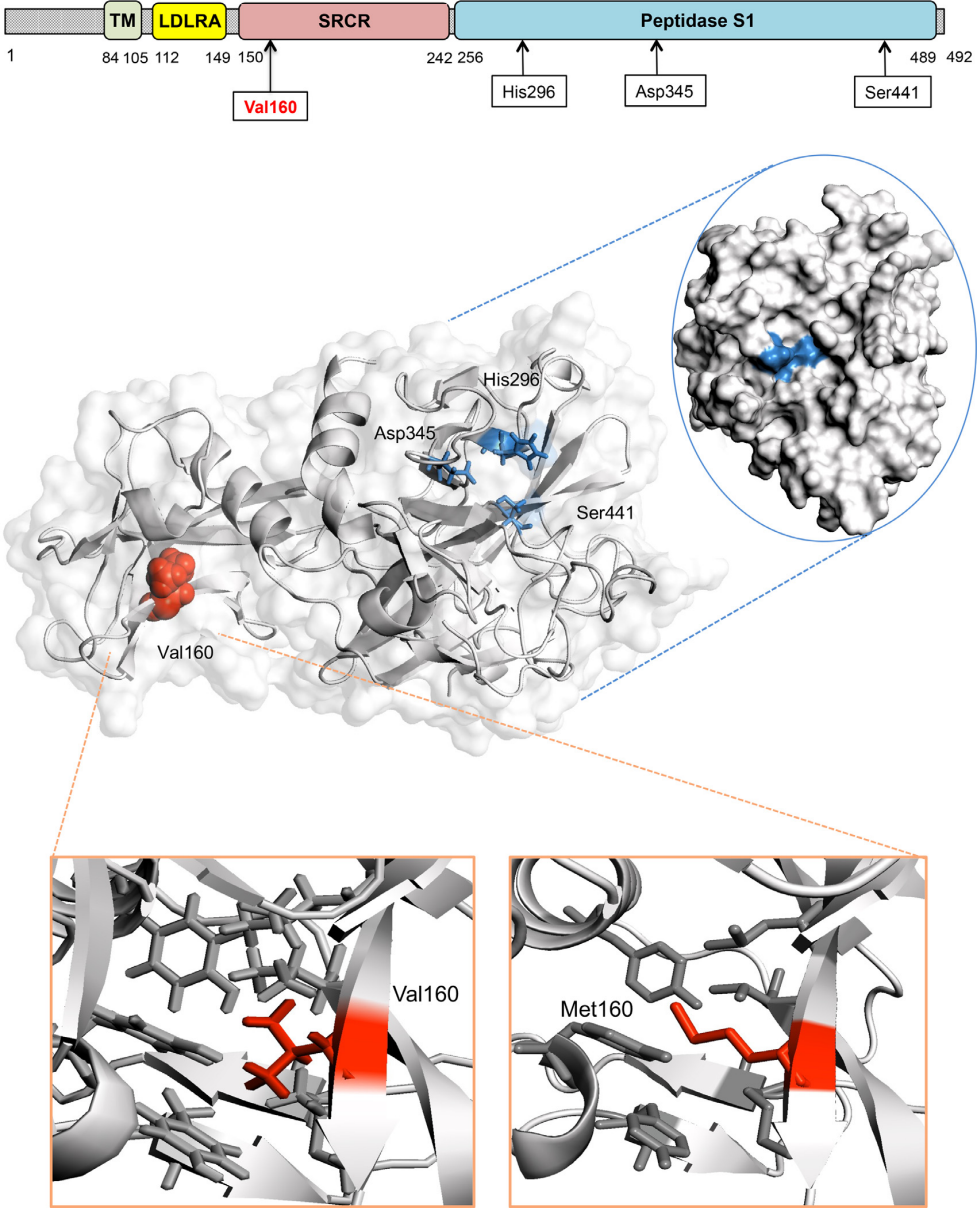
The TMPRSS2 protein and the p.Val160Met variant The TMPRSS2 protein is composed of a cytoplasmic region (residues 1–84), a transmembrane region (TM, residues 85–105) and an extracellular region (residues 106–492). The latter is composed of three domains: the LDLR class A (residues 112–149), the scavenger receptor cysteine-rich domain (SRCR) (residues 150–242) and the Peptidase S1 (residues 256–489), which contains the protease active site: residues His296, Asp345 and Ser441. The three-dimensional structure of the extracellular region residues 145–491 corresponding to domains SRCR-2 (in green) and Peptidase S1 (in blue) is presented. Valine 160 (Val 160, depicted as a red sphere on the cartoon), which harbours variant p.Val160Met, occurs in the SRCR domain and spatially far from the TMPRSS2 catalytic site (mapped onto the surface of TMPRSS2).

We extracted 377 TMPRSS2 genetic variants reported as loss of function (LoF), missense or inframe and indel in the database of population genetic variations GnomAD (v2.1.1). All variants passed quality filters in GnomAD. Nine variants flagged by GnomAD as carrying dubious annotation or quality were excluded. Forty variants were loss of function or indel and the remaining 328 were missense. All variants except for one (rs12329760 C>T) were very rare (MAF <0.001). We focused on missense variants and studied the evolutionary conservation of TMPRSS2 amino acids and the impact of amino acid substitution on TMPRSS2 protein structure (described in Methods). The first experimental structure of TMPRSS2 was released in the public domain in June 2021. Although the trypsin domain was well resolved, several unstructured loops were present in the SRCR domain. We, therefore, used homology modelling and the Phyre2 server to model the missing regions of SRCR (Fig. 1). For completeness, a comparison between the Phyre2 model and the recently released (August 2021) AlphaFold [32] model is presented in Figure S1 (Phyre2 versus AlphaFold model: root mean square deviation [RMSD] 0.5 Å). We identified the chemical and physical bonds that stabilize the TMPRSS2 structure (i.e. hydrogen bonds, cysteine and salt bridges, as detailed in the Methods) and are affected by amino acid substitutions naturally occurring in the human population. A total of 137 variants were predicted damaging to the structure and/or function of TMPRSS2. Of these, 136 variants are extremely rare in the human population, with an average minor allele frequency (MAF) of 9.67 × 10^−6^ (cumulative MAF of 7.3 × 10^−4^) and are, therefore, unlikely to be of use as a marker of COVID-19 infection severity in the general population. The remaining variant, rs12329760, (NC_000021.9:g.41480570 C>T [GRCh38.p13], p.Val160Met on Ensembl transcript ENST00000332149.5 and Val197Met on the Ensembl transcript ENST00000398585.3) is predicted damaging and causes the substitution of an evolutionary conserved valine to methionine (Figure S2). Overall, the minor allele frequency (MAF) of this variant is 0.25 in the human population, with 6.7% homozygous individuals (9,587 T/T homozygotes out of 141,456 individuals sequenced as part of the GnomAd project). Under Hardy-Weinberg equilibrium and a MAF of 0.25, it is expected that 37% of individuals will be heterozygous for this variant. The MAF of rs12329760 T varies according to ethnicities and ranges from 0.14 in Ashkenazi Jewish to 0.38 in East Asian populations (0.15 in Latino, 0.23 in non-Finnish Europeans, 0.25 in South Asians, 0.29 in African/African Americans and 0.37 in Finnish Europeans). This highly conserved valine occurs in the scavenger receptor cysteine-rich (SRCR) domain, whose function within TMPRSS2 is still not fully understood, although a role in ligand and/or protein interaction has been proposed [45]. Indeed, this domain is present in several proteins involved in host defence, such as CD5, CD6 and Complement factor I [46,47].

**Fig. 2:**
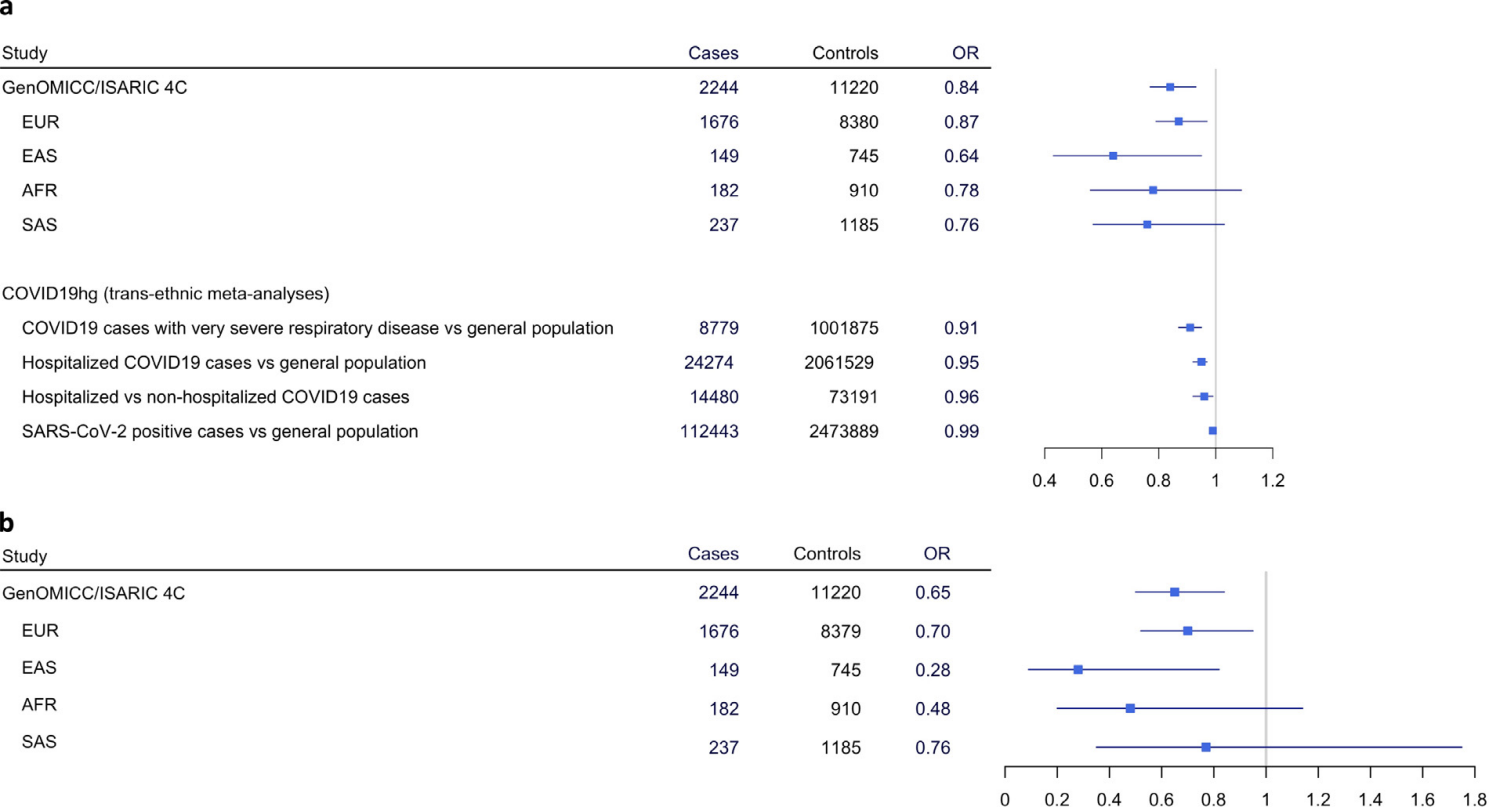
Association of TMPRSS2 rs12329760 to COVID-19 severity Results are presented for the additive (a) and the recessive (b) model using different COVID-19-positive patient cohorts. The results from large GWAS meta-analyses performed as part of the COVID-19hg initiative (1) are also shown. OR, odds ratio; EUR, European; EAS, East Asian; AFR, African; SAS, South Asian.

We first analysed the relation between TMPRSS2 rs12329760 and life-threatening SARS-CoV-2 infection in 2,244 critically ill, hospitalized, COVID-19 positive patients from 208 UK intensive care units (ICUs) (Table 1) recruited as part of the GenOMICC (genomicc.org) and ISARIC 4C (isaric4c.net) projects. These patients were representative of critically ill patients with COVID-19 in the UK population during the first Sars-Co-V2 outbreak of 2020[8]. Patients were treated in intensive care units (ICU/ITU) because of their propensity to critical respiratory failure due to COVID-19. Within the GenOMICC cohort (*n* = 2,109), mean age was 57.3 ± 12.1, 624 (30%) patients were females, and 396(19%) had comorbidities; 1,557 (74%) required invasive ventilation and 459 (22%) died within 60 days. Within the ISARIC 4C cohort (*n* = 135), mean age was 57.3 ± 2.9, 46 (34%) were females, and 40 (30%) had comorbidities; 25 (19%) required invasive ventilation and 22 (16%) died within 60 days, as described in[8]. 11,220 ancestry-matched individuals without a COVID-19-positive PCR test from the UK BioBank, acted as controls. Under an additive model, we found that the minor T allele of rs12329760 was significantly associated with a protective effect against severe COVID-19 in individuals of European ancestry (1,676 cases, 8,379 controls) with an OR of 0.87 (95%CI:0.79–0.97, *p* = 0.01). A protective effect was also observed in individuals of East Asian ancestry (149 cases, 745 controls; OR 0.64, 95%CI:0.43–0.95, *p* = 0.03). Similar effect sizes were observed in South Asians and Africans, but did not reach statistical significance, most likely as a result of the small sample size (Fig. 2). We further confirmed this protective effect on a trans-ethnic meta-analysis, using the entire cohort of 2,244 patients (OR 0.84, 95%CI:0.77–0.93, *p* = 5.8 × 10^−4^, Fig. 2, panel A). A heterogeneity analysis suggested that the T allele has a similar effect across different ethnicities (*p* = 0.47). To ascertain that this association was not an artefact due to population bias in the UK BioBank controls, the results from the European cohort were confirmed on an independent control population (45,875 unrelated individuals of European ancestry from 100 K Genomes [48]: OR 0.89, 95%CI:0.81–0.99, *p* = 0.02). Under a recessive model (i.e. individuals homozygous for the T allele), the trans-ethnic meta-analysis on 2,244 critically ill COVID-19 patients estimated an OR of 0.65 for TT homozygotes (95%CI:0.50–0.84, *p* = 1.3 × 10^−3^). In subset analyses, the OR was estimated at 0.70 (95%CI:0.52–0.95, *p* = 0.024) in Europeans, and 0.28 (95%CI:0.09–0.82, *p* = 0.019) in East Asians versus their corresponding ancestry-matched controls (Fig. 2, panel B).

**Table 1:** Characteristics of 2,244 GenOMICC/ISARIC patients and 11,220 BioBank controls included in the study.

Patient characteristics	Cases (*n* = 2,244)		Controls (*n* = 11,220)
		missing data	
Females, n. (%)	670[30]		6,075[54]
Age (years), mean ± SD	57.3 ± 11.6		66.1 ± 8.0
Invasive ventilation, n. (%)	1,582 (70.50)	66 (2.94)	n.a
Death, n. (%)	481 (21.44)	368 (16.40)	n.a
Ancestry			
European, n. (MAF)	1,676 (0.20)		8,380 (0.23)
South Asian, n. (MAF)	237 (0.21)		1,185 (0.24)
African, n. (MAF)	182 (0.26)		910 (0.29)
East Asian, n. (MAF)	149 (0.28)		745 (0.38)

MAF, minor allele frequency; n.a, not available.

To assess whether the rs12329760 could be a proxy for an association with a nearby expression quantitative trait locus (eQTL), colocalisation analysis was performed to compare the GWAS signal at the locus to eQTL associations for TMPRSS2 and neighbouring gene MX1 in GTex version 8 [44] (Figure S3 A-C), using an Approximate Bayes Factor approach [43]. Under an assumption of a single causal variant within the locus for each trait, the posterior probability of a common causal variant was 1.1% for TMPRSS2 expression and 2.0% for MX1 expression, compared to posterior probabilities of 67% and 42% respectively for independent associations. Sensitivity analysis showed that the analysis was robust to choice of prior probabilities: more stringent software-default single-trait priors increased the posterior probabilities of null or single-trait-only association hypotheses, but had little impact on the colocalisation probability (1.1% for TMPRSS2 and 0.2% for MX1); varying prior probability for colocalisation (Figure S3 D-F) had an impact only when approaching the prior for single-trait associations, and did not result in posterior probabilities for colocalisation exceeding those for separate associations. Although independent contributions from multiple variants towards the genetic association cannot be excluded, this indicates that any genetic association between rs12327960 and severe COVID-19 is unlikely to be attributable to linkage disequilibrium with an eQTL and, thus, modification of protein function is more likely.

For additional corroboration of the genetic signal, we investigated the results of large GWAS meta-analyses performed in the context of the COVID-19 Host Genetics Initiative (COVID-19hg, available at https://www.COVID-19hg.org/, release 6, June 2021) [42]. Compared with the general population, in a trans-ethnic meta-analysis, the minor T allele of rs12329760 was associated with a significantly protective effect against severe COVID-19 (patients requiring hospitalization for COVID-19): 24,274 cases versus 2,061,529 controls; OR 0.95, 95%CI:0.92–0.97, *p* = 4.72 × 10^−6^. In ancestry-specific sub-group analyses, this effect was significant for a European population (OR 0.94, 95%CI:0.91–0.96, *p* = 5.66 × 10^−6^), but not for individuals of African, Hispanic-American, or admixed African/Hispanic/American ancestry; however, lower sample sizes for these groups limited study power, and subgroup analyses were not available for Asian populations. The protective effect was particularly evident in confirmed, critically ill cases (8,779 cases versus 1,001,875 population controls; OR 0.91, 95%CI:0.87–0.95, *p* = 8.18 × 10^−6^). Furthermore, the rs12329760 T allele was associated with reduced risk of hospitalisation after confirmed infection (14,480 hospitalised versus 73,191 cases not requiring hospitalization within 21 days after the test): OR 0.96, 95%CI:0.92–0.99, *p* = 0.012. Finally, there was no significant difference (*p* = 0.056) in the prevalence of the T allele between the general population (*n* = 2,473,889) and pooled individuals with a laboratory-confirmed SARS-CoV-2 infection (including hospitalized and life-threatening COVID-19 cases from the metanalyses previously described) or with a self-reported or physician-confirmed COVID diagnosis (total *n* = 112,443 cases, Fig. 2, panel A).

Although these meta-analyses include UK Biobank data and all except the hospitalised versus non-hospitalised comparison include data from the GenOMICC/ISARIC 4C cohort, and thus do not provide completely independent replication, these cohorts only comprise less than 25% of the total cases, limiting the impact of this single study on the overall results. These data therefore provide further support for our hypothesis that the TMPRSS2 rs12329760 variant has a protective effect against severe and/or life-threatening COVID-19. However, studies examining the prevalence of this variant in SARS-CoV-2 infected asymptomatic or pauci-symptomatic individuals are needed to ascertain its protective effect against mild viral infection.

**Fig. 3:**
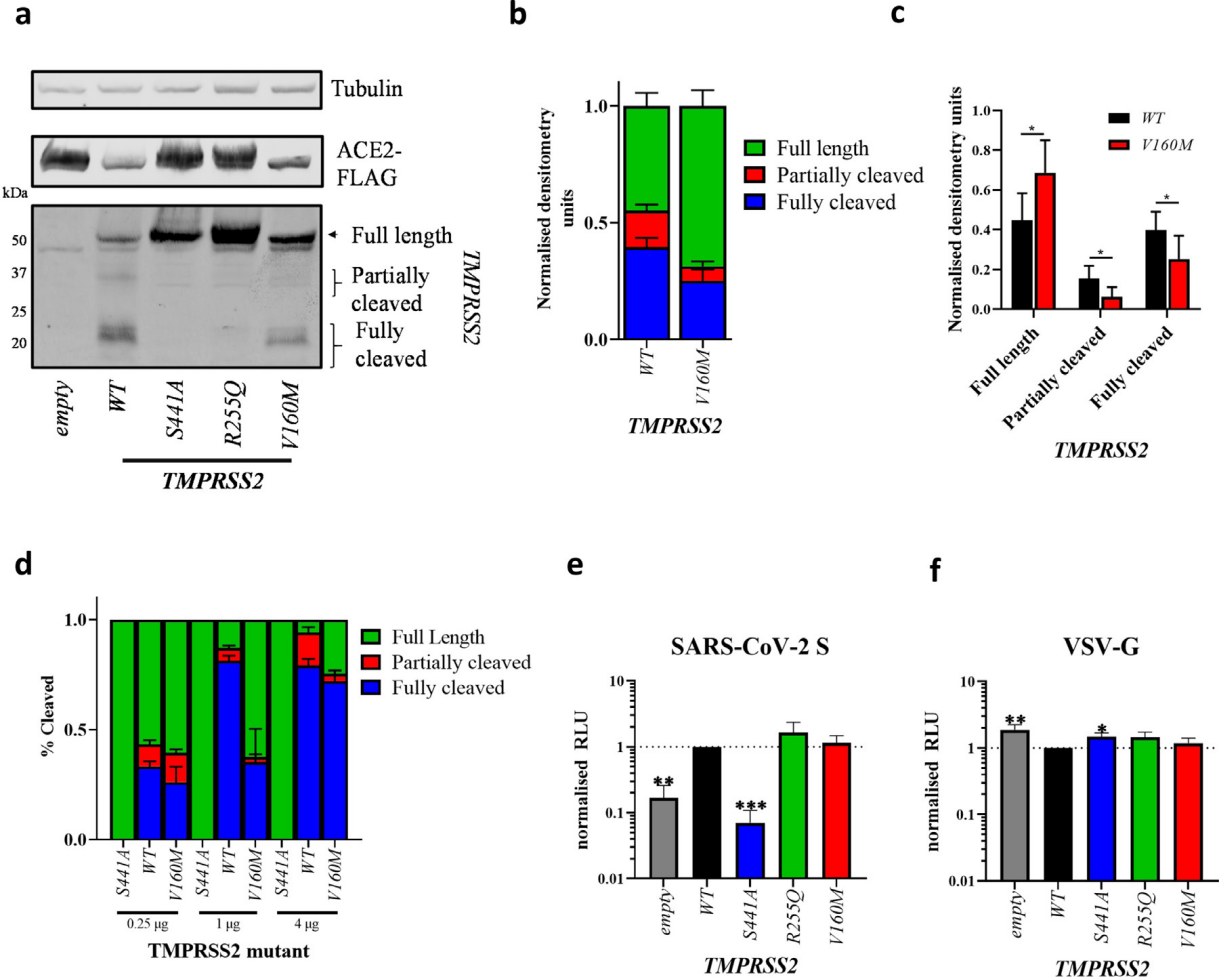
Phenotypic impact of the TMPRSS2 V160M variant on autocleavage and SARS-CoV-2 spike-mediated entry; (A) Western blot analysis of TMPRSS2 autocleavage after expression in HEK 293Ts. (B,C, D) densitometry was determined in ImageJ and shows mean±standard deviation from *N* = 6 (B,C) or *N* = 3 (D) independent repeats. Statistics determined by two-tailed Student's *t*-test. Entry of lentiviral pseudotypes expressing (E) SARS-CoV-2 spike glycoprotein or (F) Vesicular stomatitis virus glycoprotein (VSV-G) into HEK 293Ts co-expressing ACE2-FLAG and either empty vector or TMPRSS2 variants. Data shows mean±standard deviation of 3 independent repeats from different weeks, normalised to WT TMPRSS2. (E,F) Statistics determined by one-way ANOVA with multiple comparisons against WT on Log-transformed data (after determining log normality by the Shapiro-Wilk test and QQ plot). Values in µg indicate the amount of TMPRSS2 plasmid added to each condition. RLU, relative luminescence units. *, 0.05 ≥ *P* > 0.01; **, 0.01 ≥ *P* > 0.001, ***, 0.001 ≥ P*.*

To investigate the phenotypic effect of the TMPRSS2 V160M variant, we co-transfected 293Ts cells, which we have previously confirmed that they do not endogenously express ACE2 or TMPRSS2 [24], with ACE2 and either TMPRSS2 wild type (TMPRSS2_WT_) or V160M (TMPRSS2_V160M_), as previously described [24]. We and others previously observed that co-expression of TMPRSS2 and ACE2 results in rapid cleavage of ACE2. We, therefore, used a mutant ACE2 that is more poorly degraded by TMPRSS2[49]. Two additional TMPRSS2 variants were included as controls: the catalytically inactive S441A (TMPRSS2_S441A_) and the catalytically active R255Q (TMPRSS2_R255Q_), that is unable to autocleave[16]. First, we investigated the autocleavage pattern of the different TMPRSS2 variants. The N-terminal membrane-bound part of TMPRSS2 can exist as different cleaved intermediates: a full-length uncleaved form of approximately 55 kDa, a partially cleaved form, and a fully cleaved form of 20 kDa. The latter is the product of TMPRSS2 autocleavage at arginine 255, which results in the liberation of the catalytically active protease domain in the extracellular space, leaving a small transmembrane N-terminal domain [16]. Wild type TMPRSS2 is expressed as roughly equal amounts of full-length and fully cleaved forms, with a small amount of partially cleaved product. As expected, the catalytically inactive TMPRSS2_S441A_ and the non-autocleavable TMPRSS2_R255Q_ resulted in only the full-length TMPRSS2 being expressed. However, TMPRSS2_V160M_ resulted in a significantly higher proportion of full-length (55 kDa), and a significantly lower proportion of fully cleaved protein (20 kDa) (*p* < 0.05, Student's *t*-test). This difference was clear across a range of TMPRSS2 concentrations, with TMPRSS2 showing a concentration-dependant autocleavage phenotype: the higher the concentration of TMPRSS2, the higher the amount of autocleavage. Overall, these data suggest the V160M substitution exerts a partial inhibitory effect on the proteolytic autocleavage of TMPRSS2 (see Fig. 3A-D, Supplementary Figure S4).

Subsequently, we investigated the effect of TMPRSS2_V160M_ on promoting viral entry, using a previously described SARS-CoV-2 pseudovirus entry assay [24]. Pseudovirus expressing the glycoprotein from the vesicular stomatitis virus (VSV-G) was used as a control, as this virus enters cells in a TMPRSS2-independent manner [24]. Briefly, cells co-transfected with ACE2 and TMPRSS2 wild type or variants were incubated with the pseudovirus (as described in [24,38]) and, after 48 h, luminescence was measured. TMPRSS2_WT_ enhanced viral entry by ∼5-fold compared to empty vector, while the catalytically dead TMPRSS2_S441A_ showed no enhancement (Fig. 3E). The non-autocleavable mutant TMPRSS2_R255Q_ showed similar enhancement, suggesting that autocleavage is dispensable for optimal TMPRSS2-mediated enhancement. TMPRSS2_V160M_ showed no significant difference in viral entry compared to the TMPRSS2_WT_. Overall, the expression of catalytically active TMPRSS2 proteins only slightly inhibited VSV-G mediated entry (Fig. 3F).

**Fig. 4:**
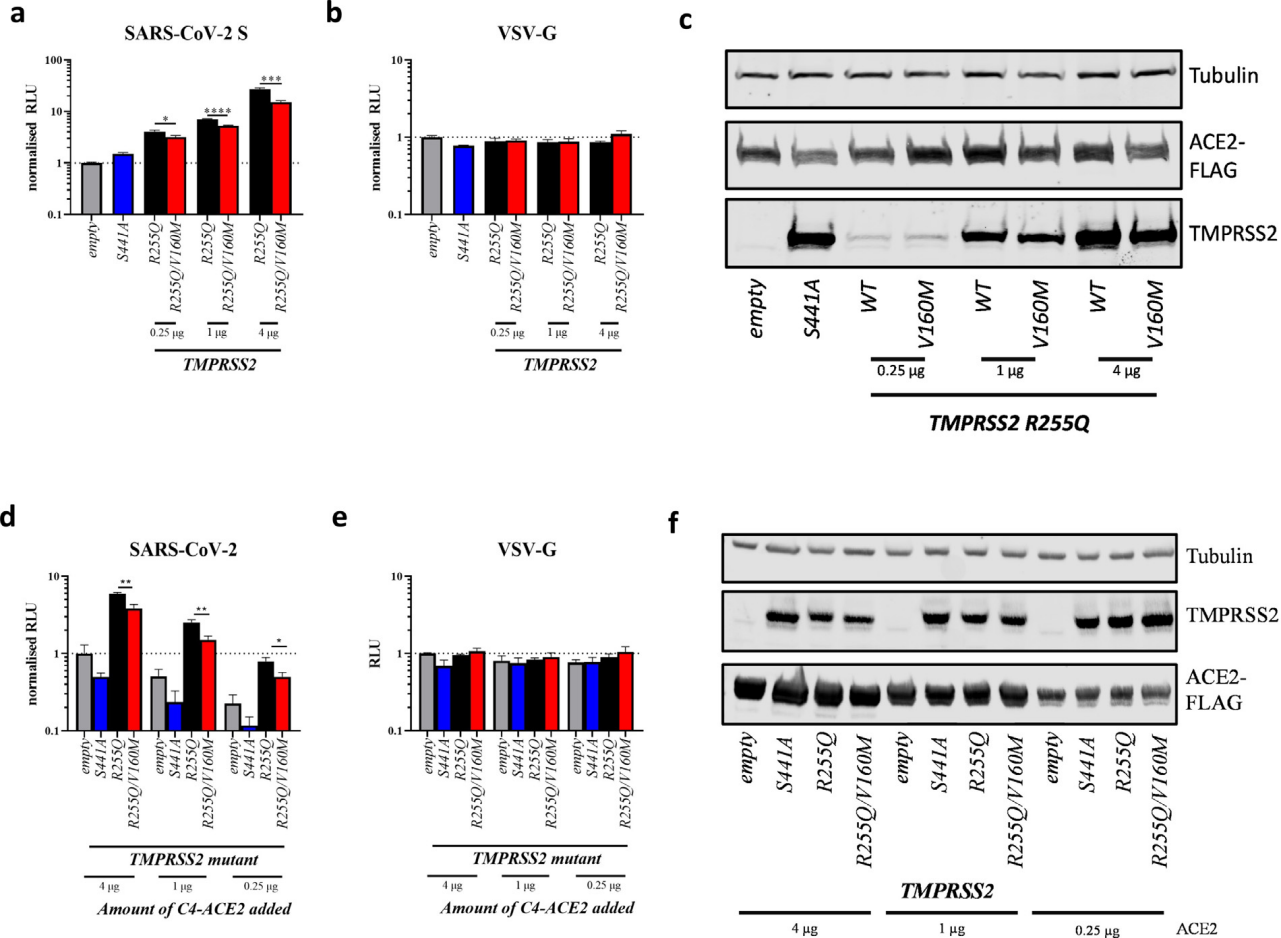
Phenotypic impact of the TMPRSS2 non-autocleavable version of the V160M variant on SARS-CoV-2 spike-mediated entry Entry of lentiviral pseudotypes expressing (A,D) SARS-CoV-2 spike glycoprotein or (B,E) vesicular stomatitis virus glycoprotein (VSV-G) into HEK 293Ts co-expressing ACE2-FLAG and either empty vector or TMPRSS2 variants. Data shows mean±standard deviation of 3 independent repeats from different weeks, normalised to empty vector. A-C shows titrations of mutant TMPRSS2 with constant ACE2 expression, while D-E show titrations of ACE2 with constant levels of TMPRSS2 expressed. (A,B,D,E) statistics determined by two-tailed Student's *t*-test. (C,F) Western blot analysis of TMPRSS2 autocleavage mutant (R255Q) titration with or without the V160M substitution. µg values indicate the amount of TMPRSS2 or ACE2 plasmid added to each condition. RLU, relative luminescence units. *, 0.05 ≥ *P* > 0.01; **, 0.01 ≥ *P* > 0.001, ***, 0.001 ≥ P*.*

The partial inhibitory effect exerted by the V160M variant on the proteolytic autocleavage of TMPRSS2 resulted in a far greater proportion of uncleaved, surface-expressed TMPRSS2_V160M_ compared to TMPRSS2_WT_. We compared this autocleavage seen in transfected 293T cells, to that seen in several epithelial cell lines that naturally express endogenous ACE2 and TMPRSS2 [24]: the human lung cell line, Calu-3, and the human colorectal adenocarcinoma cell line, Caco-2, both of which are extensively used for SARS-CoV-2 research. Interestingly, no fully cleaved TMPRSS2 could be detected as opposed to 293T cells, while both cell lines expressed mostly full length or partially cleaved TMPRSS2. This again suggests that the high levels of autocleavage seen in 293T cells may be, in part, an artefact of overexpression (Supplementary Figure S5). Therefore, we re-assessed whether TMPRSS2_V160M_ affects SARS-CoV-2 S-expressing pseudovirus entry by using the double mutant TMPRSS2_R255Q/V160M_ (which does not autocleave and is, therefore, more similar to endogenous TMPRSS2 in Calu-3 and Caco-2 cells), to control for protein cell-surface expression. Under these conditions, and across a range of plasmid titrations of both TMPRSS2 mutants and ACE2, TMPRSS2_R255Q/V160M_ showed a significantly reduced ability to promote SARS-CoV-2 S-expressing pseudovirus compared to TMPRSS2_R255Q_ alone, despite equal protein expression (Fig. 4A,C,D,F). Again, TMPRSS2_R255Q/V160M_ had no effect on VSV-G-mediated entry (Fig. 4B,E).

## Discussion

Overall, our results suggest that the rs12329760 C>T variant results in a moderately less catalytically active TMPRSS2, which is less able to autocleave and prime the SARS-CoV-2 spike protein. This may explain the protective effect against life-threatening COVID-19 observed in our cohort of patients admitted to ICU, compared to the general population. Such an effect was more prominent in homozygotes (recessive model) for the rs12329760 C>T in whom a 30% (OR 0.70) risk reduction was observed. Unfortunately, we did not have samples from asymptomatic/pauci symptomatic patients, but data from COVID-19hg meta-analyses appear to suggest that the rs12329760 variant has no protective effect against SARS-Co-V2 infection per se.

The allele frequency of TMPRSS2 rs12329760 (data from GnomAD population database) varies across different populations and is higher in East Asian and Finnish individuals (MAF 0.38 and 0.37, respectively) compared to south Asians (MAF 0.25) and non-Finnish Europeans (MAF 0.23). The lowest frequency of the T allele is reported in Latino and Jewish-Ashkenazi individuals (MAF 0.15). However, in our study, the small sample size of populations of non-European ancestry does not allow conclusions on the effect size of TMPRSS2 rs12329760 in different ethnicities. Genotyping of the TMPRSS2 rs12329760 variant on large COVID19 cohorts of patients of non-European genetic ancestry is, therefore, needed to assess its role in determining the differences in the severity of COVID-19 across various populations (e.g. between East Asia and Europe [50]). Indeed, a recent study showed a lower T allele frequency in a small cohort of Chinese patients with life-threatening COVID-19 compared to the population frequency [51]. Although the differences in the proportion of SARS-CoV-2 patients who develop severe COVID-19 across different populations [50] are more likely to be explained by social behaviour, public health measures to curb outbreaks, exposure to other viruses and immunological factors, human genetic variation across different populations may also contribute to the observed differences.

The pharmacological inhibition of TMPRSS2 using serine protease inhibitors, such as camostat and nafamostat, has been proposed as a pharmacological treatment of COVID-19 patients. In vitro [12] and animal studies have demonstrated that camostat can block viral entry (reviewed in [52]), and initial reports on the repurposing of camostat in COVID-19 patients have provided promising results [53]. However, a recently completed clinical trial using camostat in patients hospitalized for severe COVID-19, did not demonstrate a significant reduction in time-to-clinical improvement compared to placebo [54]. As the authors suggest, these patients were likely to have passed the most active stage of viral replication at the time of treatment and were in the hyper-inflammatory stage of the COVID-19, thus possibly explaining the lack of camostat efficacy. Several additional clinical trials of camostat in COVID-19 are currently underway [29]. Recently, the placebo-controlled phase III trial conducted in Japan on pauci symptomatic COVID-19 patients administering camostat mesilate 600 mg 4 times a day did not meet its primary end point of time to negative Sars-CoV-2 test [55], however data on secondary end points, such as progression to severe or life-threatening COVID-19, are still not publicly available.

Very little is known on TMPRSS2 and further extensive in vitro and in vivo studies on its pathophysiology are necessary. Since the beginning of the COVID-19 pandemic, the interest in TMPRSS2 has focused only on its role as a serine protease involved in the activation of the SARS-CoV-2 spike protein. However, as a soluble protease, TMPRSS2 may have additional substrates, and in vitro studies have demonstrated that PAR2 is one of these substrates [56,57]. PAR2 is expressed in several tissues, including lung, vascular endothelial and vascular smooth muscle cells [58,59] and its protease-mediated activation promotes inflammation by inducing prostaglandin synthesis and cytokine production in the lungs and other organs [60], [61], [62], [63], [64]. An intriguing hypothesis is that, similar to other soluble serine proteases, such as the human airway trypsin-like protease HAT (also known as TMPRSS11D), the soluble wild type TMPRSS2 protease may also have a role in promoting inflammation in the lungs and other tissues.

Since May 2020, when we reported the TMPRSS2 variant rs12329760 as possibly damaging to protein structure/function and raised the possibility that it could partly explain host susceptibility to COVID-19 severity [65], several other studies have also supported this hypothesis [66], [67], [68], [69], [70], [71] . In this study we have confirmed our initial hypothesis and provided a mechanistic effect to explain how this variant may contribute to the host susceptibility to severe COVID-19.

As previously discussed, one limitation of our study was the lack of access to a cohort of asymptomatic/pauci symptomatic COVID-19 patients. In the absence of such a cohort, we considered the general population as a good proxy and used this for comparison with COVID-19 severe cases. Indeed, a recent systematic review and metanalysis shows that one third of COVID-19 positive cases do not develop symptoms [72]. When well-characterized cohorts of asymptomatic/pauci symptomatic COVID-19 patients become available, it will be possible to further investigate the role of TMPRSS2 variant rs12329760 on Sars-Co-V2 infection. Another limitation of this study is that we did not directly validate our results in endogenously expressing cell lines, such as like Calu-3, as this would require gene editing the endogenous TMPRSS2. Calu-3 cells are extremely slow growing and highly resistant to single cell cloning, thus making this cell line not particularly suitable for gene editing.

In conclusion, the T allele of the common TMPRSS2 variant rs12329760 confers a reduced risk of severe COVID-19. Similar to what observed in the TMPRSS2 KO mouse, the Val160Met substitution, which exerts a partial inhibitory effect on the proteolytic autocleavage of TMPRSS2 and the priming of the SARS-CoV-2 spike protein, is associated with a milder COVID-19 infection compared to the wild type. Differences in population frequency of this genetic variant may contribute to the reported variability in COVID-19 severity across various ethnicities and studies on large COVID-19 cohorts of patients of non-European genetic ancestry are needed to clarify this. Further studies are needed to assess the expression of TMPRSS2 across different age groups; indeed a reduced TMPRSS2 expression in younger compared to older individuals, as observed in mice and in preliminary human studies, could help explain age-related differences in COVID-19 morbidity. Moreover, TMPRSS2 could be a viable drug target in COVID-19 patients, and camostat mesilate, or other novel TMPRSS2 inhibitors, may have a role in the treatment of COVID-19. Clinical trials are needed to confirm this.

## Funding

Wellcome Trust, BBSRC, UKRI Future Leader's Fellowship, Health Data Research UK

AD and NP were supported by the 10.13039/100010269Wellcome Trust (grants 104,955/Z/14/Z, 218,242/Z/19/Z and 211,496/Z/18/Z) and TK by the BBSRC (grants BB/P011705/1 and BB/P023959/1), VSS is supported by UKRI Future Leader's Fellowship (MR/S032304/1), J-LC is supported by 10.13039/100000011Howard Hughes Medical Institute, Rockefeller University, St. Giles Foundation, Fisher centre for Alzheimer's Research Foundation, Meyer Foundation, Square Foundation, Grandir - Fonds de solidarité pour l'enfance, SCOR Corporate Foundation for Science, Institut National de la Santé et de la Recherche Médicale (INSERM), University of Paris, National Institutes of Health (R01AI088364), French Foundation for Medical Research (EQU201903007798), FRM and French National Research Agency (ANR) GENCOVID project (ANR-20-COVI-0003); LA is supported by the 10.13039/501100001665Agence Nationale de la Recherche (ANR-10-IAHU-01, ANR-10-LABX-62-IBEID), TPP and WSB are supported by 10.13039/501100000268BBSRC grants BB/R013071/1 and BBSRC and the G2P-UK National Virology consortium (funded by MRC/UKRI, grant ref: MR/W005611/1), AT was supported by Roslin Institute Strategic Programme Grants from the BBSRC (BBS/E/D/10,002,070 and BBS/E/D/30,002,275) and Health Data Research UK (references HDR-9004 and HDR-9003).

## Author contributions

NP, EP-C, AT and JKB contributed to population data analysis. AD, TK and MJES contributed to 3D modelling and structural analysis. TPP and WSB contributed to laboratory work. NP, EP-C, TPP and AD contributed to data analysis. NP, TPP, WSB, AD contributed to study design. NP, TPP, EP-C, AC, VS-S, J-LC, LA, WSB, JKB, MJES and AD contributed to interpretation of findings and manuscript preparation. AD conceived the study, contributed to study coordination and wrote the first draft of the manuscript. All authors approved the final version of the manuscript.

## Data availability

Full summary-level data in support of the findings of this study are available for download from https://genomicc.org/data. Individual level data can be analysed by qualified researchers in the ISARIC 4C/GenOMICC data analysis platform by application at https://genomicc.org/data. BioBank data and Genomics England data are available to registered researchers at https://www.ukbiobank.ac.uk/ and https://www.genomicsengland.co.uk/. The COVID-19 Host Genetics Initiative2 (COVID-19hg) summary statistics are available at https://www.COVID-19hg.org/.

Supplementary Information is available for this paper.

## Declaration of Competing Interest

Dr. David reports grants from Wellcome Trust during the conduct of the study; Dr. Parkinson reports grants from Wellcome Trust during the conduct of the study; Dr. Peacock reports grants from MRC/UKRI, grants from BBSRC during the conduct of the study; Dr. Pairo-Castineira has nothing to disclose. Dr. Khanna reports grants from BBSRC during the conduct of the study; Dr. Cobat has nothing to disclose. Dr. Tenesa reports grants from BBSRC, grants from Health Data Research UK during the conduct of the study. Dr. Sancho-Shimizu reports grants from UKRI Future Leader's Fellowship during the conduct of the study; Dr. Casanova reports other from Howard Hughes Medical Institute, other from Rockefeller University, other from St. Giles Foundation, other from Fisher centre for Alzheimer's Research Foundation, other from Meyer Foundation, other from Square Foundation, other from Grandir - Fonds de solidarité pour l'enfance, other from SCOR Corporate Foundation for Science, other from Institut National de la Santé et de la Recherche Médicale (INSERM), other from University of Paris, other from National Institutes of Health (NIH), other from French Foundation for Medical Research (FRM), other from FRM and French National Research Agency (ANR) GENCOVID project during the conduct of the study; Dr. Abel reports other from Agence Nationale de la Recherche during the conduct of the study; Dr. Barclay reports grants from BBSRC during the conduct of the study; Dr. Baillie has nothing to disclose. Dr. Sternberg reports grants from Wellcome Trust, grants from BBSRC, during the conduct of the study.

## Appendix 2: ISARIC4C Investigators

Consortium Lead Investigator: J Kenneth Baillie.

Chief Investigator: Malcolm G Semple.

Co-Lead Investigator: Peter JM Openshaw.

ISARIC Clinical Coordinator: Gail Carson.

Co-Investigator: Beatrice Alex, Petros Andrikopoulos, Benjamin Bach, Wendy S Barclay, Debby Bogaert, Meera Chand, Kanta Chechi, Graham S Cooke, Ana da Silva Filipe, Thushan de Silva, Annemarie B Docherty, Gonçalo dos Santos Correia, Marc-Emmanuel Dumas, Jake Dunning, Tom Fletcher, Christoper A Green, William Greenhalf, Julian L Griffin, Rishi K Gupta, Ewen M Harrison, Julian A Hiscox, Antonia Ying Wai Ho, Peter W Horby, Samreen Ijaz, Saye Khoo, Paul Klenerman, Andrew Law, Matthew R Lewis, Sonia Liggi, Wei Shen Lim, Lynn Maslen, Alexander J Mentzer, Laura Merson, Alison M Meynert, Shona C Moore, Mahdad Noursadeghi, Michael Olanipekun, Anthonia Osagie, Massimo Palmarini, Carlo Palmieri, William A Paxton, Georgios Pollakis, Nicholas Price, Andrew Rambaut, David L Robertson, Clark D Russell, Vanessa Sancho-Shimizu, Caroline J Sands, Janet T Scott, Louise Sigfrid, Tom Solomon, Shiranee Sriskandan, David Stuart, Charlotte Summers, Olivia V Swann, Zoltan Takats, Panteleimon Takis, Richard S Tedder, AA Roger Thompson, Emma C Thomson, Ryan S Thwaites, Lance CW Turtle, Maria Zambon.

Project Manager: Hayley Hardwick, Chloe Donohue, Fiona Griffiths, Wilna Oosthuyzen.

Project Administrator: Cara Donegan, Rebecca G. Spencer.

Data Analyst: Lisa Norman, Riinu Pius, Thomas M Drake, Cameron J Fairfield, Stephen R Knight, Kenneth A Mclean, Derek Murphy, Catherine A Shaw.

Data and Information System Manager: Jo Dalton, Michelle Girvan, Egle Saviciute, Stephanie Roberts, Janet Harrison, Laura Marsh, Marie Connor, Sophie Halpin, Clare Jackson, Carrol Gamble, Daniel Plotkin, James Lee.

Data Integration and Presentation: Gary Leeming, Andrew Law, Murray Wham, Sara Clohisey, Ross Hendry, James Scott-Brown.

Material Management: Victoria Shaw, Sarah E McDonald.

Patient Engagement: Seán Keating.

Outbreak Laboratory Staff and Volunteers: Katie A. Ahmed, Jane A Armstrong, Milton Ashworth, Innocent G Asiimwe, Siddharth Bakshi, Samantha L Barlow, Laura Booth, Benjamin Brennan, Katie Bullock, Benjamin WA Catterall, Jordan J Clark, Emily A Clarke, Sarah Cole, Louise Cooper, Helen Cox, Christopher Davis, Oslem Dincarslan, Chris Dunn, Philip Dyer, Angela Elliott, Anthony Evans, Lorna Finch, Lewis WS Fisher, Terry Foster, Isabel Garcia-Dorival, Philip Gunning, Catherine Hartley, Rebecca L Jensen, Christopher B Jones, Trevor R Jones, Shadia Khandaker, Katharine King, Robyn T. Kiy, Chrysa Koukorava, Annette Lake, Suzannah Lant, Diane Latawiec, Lara Lavelle-Langham, Daniella Lefteri, Lauren Lett, Lucia A Livoti, Maria Mancini, Sarah McDonald, Laurence McEvoy, John McLauchlan, Soeren Metelmann, Nahida S Miah, Joanna Middleton, Joyce Mitchell, Shona C Moore, Ellen G Murphy, Rebekah Penrice-Randal, Jack Pilgrim, Tessa Prince, Will Reynolds, P. Matthew Ridley, Debby Sales, Victoria E Shaw, Rebecca K Shears, Benjamin Small, Krishanthi S Subramaniam, Agnieska Szemiel, Aislynn Taggart, Jolanta Tanianis-Hughes, Jordan Thomas, Erwan Trochu, Libby van Tonder, Eve Wilcock, J. Eunice Zhang, Lisa Flaherty, Nicole Maziere, Emily Cass, Alejandra Doce Carracedo, Nicola Carlucci, Anthony Holmes, Hannah Massey.

Edinburgh Laboratory Staff and Volunteers: Lee Murphy, Sarah McCafferty, Richard Clark, Angie Fawkes, Kirstie Morrice, Alan Maclean, Nicola Wrobel, Lorna Donnelly, Audrey Coutts, Katarzyna Hafezi, Louise MacGillivray, Tammy Gilchrist.

Local Principal Investigators: Kayode Adeniji, Daniel Agranoff, Ken Agwuh, Dhiraj Ail, Erin L. Aldera, Ana Alegria, Sam Allen, Brian Angus, Abdul Ashish, Dougal Atkinson, Shahedal Bari, Gavin Barlow, Stella Barnass, Nicholas Barrett, Christopher Bassford, Sneha Basude, David Baxter, Michael Beadsworth, Jolanta Bernatoniene, John Berridge, Colin Berry, Nicola Best, Pieter Bothma, David Chadwick, Robin Brittain-Long, Naomi Bulteel, Tom Burden, Andrew Burtenshaw, Vikki Caruth, David Chadwick, Duncan Chambler, Nigel Chee, Jenny Child, Srikanth Chukkambotla, Tom Clark, Paul Collini, Catherine Cosgrove, Jason Cupitt, Maria-Teresa Cutino-Moguel, Paul Dark, Chris Dawson, Samir Dervisevic, Phil Donnison, Sam Douthwaite, Andrew Drummond, Ingrid DuRand, Ahilanadan Dushianthan, Tristan Dyer, Cariad Evans, Chi Eziefula, Chrisopher Fegan, Adam Finn, Duncan Fullerton, Sanjeev Garg, Sanjeev Garg, Atul Garg, Effrossyni Gkrania-Klotsas, Jo Godden, Arthur Goldsmith, Clive Graham, Elaine Hardy, Stuart Hartshorn, Daniel Harvey, Peter Havalda, Daniel B Hawcutt, Maria Hobrok, Luke Hodgson, Anil Hormis, Michael Jacobs, Susan Jain, Paul Jennings, Agilan Kaliappan, Vidya Kasipandian, Stephen Kegg, Michael Kelsey, Jason Kendall, Caroline Kerrison, Ian Kerslake, Oliver Koch, Gouri Koduri, George Koshy, Shondipon Laha, Steven Laird, Susan Larkin, Tamas Leiner, Patrick Lillie, James Limb, Vanessa Linnett, Jeff Little, Mark Lyttle, Michael MacMahon, Emily MacNaughton, Ravish Mankregod, Huw Masson, Elijah Matovu, Katherine McCullough, Ruth McEwen, Manjula Meda, Gary Mills, Jane Minton, Mariyam Mirfenderesky, Kavya Mohandas, Quen Mok, James Moon, Elinoor Moore, Patrick Morgan, Craig Morris, Katherine Mortimore, Samuel Moses, Mbiye Mpenge, Rohinton Mulla, Michael Murphy, Megan Nagel, Thapas Nagarajan, Mark Nelson, Lillian Norris, Matthew K. O'Shea, Igor Otahal, Marlies Ostermann, Mark Pais, Carlo Palmieri, Selva Panchatsharam, Danai Papakonstantinou, Hassan Paraiso, Brij Patel, Natalie Pattison, Justin Pepperell, Mark Peters, Mandeep Phull, Stefania Pintus, Jagtur Singh Pooni, Tim Planche, Frank Post, David Price, Rachel Prout, Nikolas Rae, Henrik Reschreiter, Tim Reynolds, Neil Richardson, Mark Roberts, Devender Roberts, Alistair Rose, Guy Rousseau, Bobby Ruge, Brendan Ryan, Taranprit Saluja, Matthias L Schmid, Aarti Shah, Prad Shanmuga, Anil Sharma, Anna Shawcross, Jeremy Sizer, Manu Shankar-Hari, Richard Smith, Catherine Snelson, Nick Spittle, Nikki Staines, Tom Stambach, Richard Stewart, Pradeep Subudhi, Tamas Szakmany, Kate Tatham, Jo Thomas, Chris Thompson, Robert Thompson, Ascanio Tridente, Darell Tupper-Carey, Mary Twagira, Nick Vallotton, Rama Vancheeswaran, Lisa Vincent-Smith, Shico Visuvanathan, Alan Vuylsteke, Sam Waddy, Rachel Wake, Andrew Walden, Ingeborg Welters, Tony Whitehouse, Paul Whittaker, Ashley Whittington, Padmasayee Papineni, Meme Wijesinghe, Martin Williams, Lawrence Wilson, Sarah Cole, Stephen Winchester, Martin Wiselka, Adam Wolverson, Daniel G Wootton, Andrew Workman, Bryan Yates, Peter Young.

## Appendix Supplementary materials

Image, application 1
