# Supplementary material for: A common TMPRSS2 variant has a protective effect against severe COVID-19
Source: Curr Res Transl Med. 2022 May;70(2):None. doi: 10.1016/j.retram.2022.103333 (PMC8743599; doi:10.1016/j.retram.2022.103333)

**Supplementary material**

**A common TMPRSS2 variant has a protective effect against severe COVID-19**

Alessia David, Nicholas Parkinson, Thomas P Peacock, Erola Pairo-Castineira, Tarun Khanna, Aurelie Cobat, Albert Tenesa, Vanessa Sancho-Shimizu, GenOMICC Investigators, ISARIC4C Investigators, Jean-Laurent Casanova, Laurent Abel, Wendy S. Barclay, J. Kenneth Baillie, Michael JE Sternberg

**Supplementary Figures**

Figure S1 page 2

Figure S2 page 3

Figure S3 page 4

Figure S4 page 5

Figure S5 page 6

**Supplementary Figure S1. AlphaFold versus Phyre2 model.**

The AlphaFold model (presented in orange) is superposed to the Phyre2 generated model (presented in deepteal). Amino acids modelled by AlphaFold at low confidence, defined by the per-residue confidence score (pLDDT) <70, are presented in light grey. The root mean square deviation (RMSD) between the AlphaFold and Phyre2 model is 0.5Å.


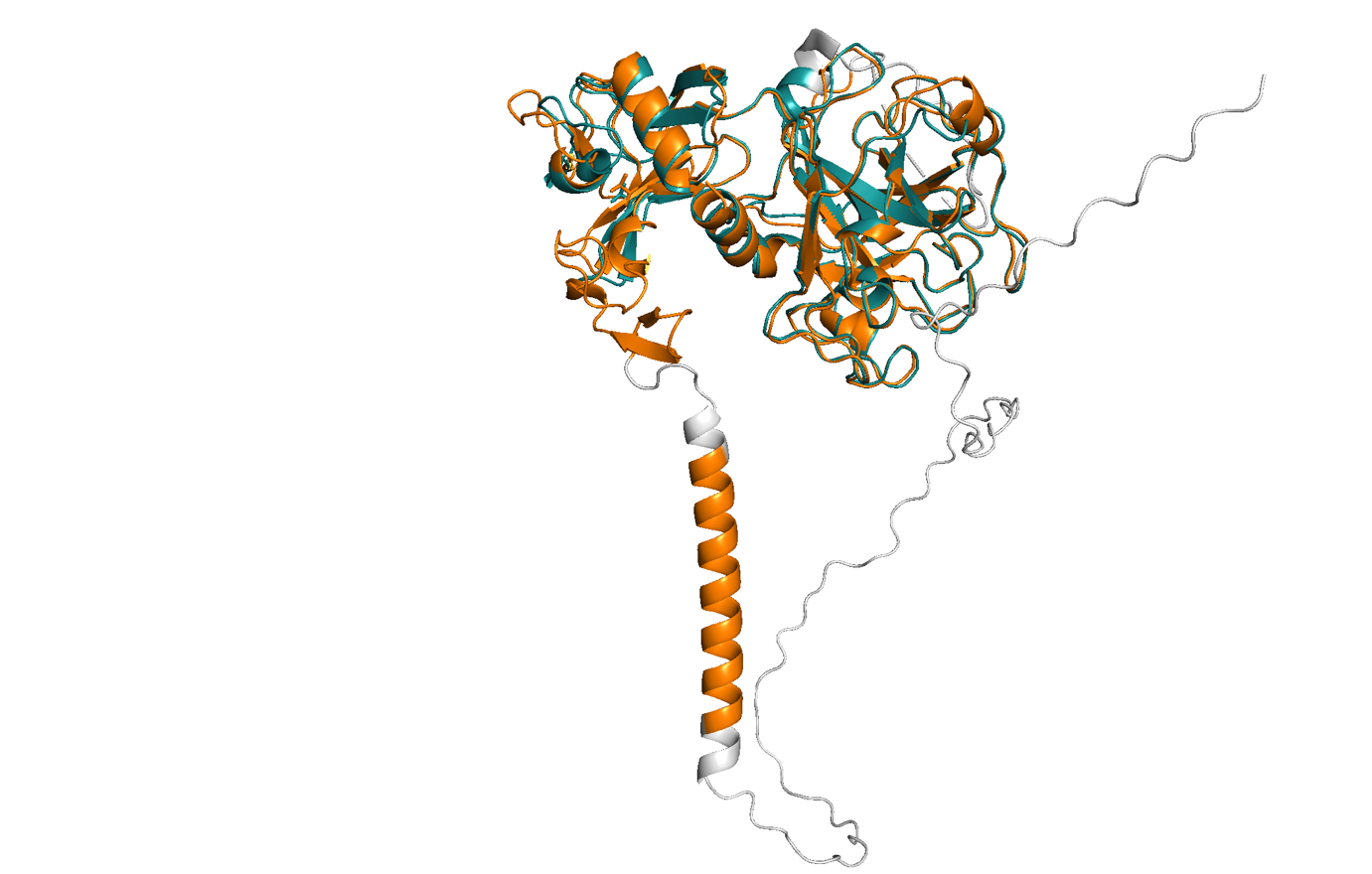


**Supplementary Figure S2.** **Multiple Sequence Alignment**. The position of the conserved TMPRSS2 residue 160 is highlighted with a red arrow. Human TMPRSS2 is identified as “QUERY”.

**Supplementary Figure S3.** **Lack of colocalisation of GWAS and eQTL signals.** A-C: Manhattan plots of GWAS associations with severe COVID-19 (A) and GTex lung eQTL associations for *TMPRSS2* (B) and *MX1* (C) in individuals of European ancestry, in the locus of the *TMPRSS2* gene ± 5kb. The dashed line shows the threshold of nominal significance (p<0.05). D-F: Colocalisation sensitivity analysis across a relevant range of priors: D shows changes in hypothesis-level prior probabilities as the SNP-level prior probability for simultaneous association with both traits (‘p12’) is varied, while E and F show the resulting posterior probabilities for *TMPRSS2* and *MX1* respectively. The competing hypotheses are defined as follows: H0 – no causal variant; H1 – causal variant for GWAS only; H2 – causal variant for eQTL association only; H3 – different causal variants for each trait; H4 – single common causative variant.

**Supplementary Figure S4. Autocleavage phenotype of different TMPRSS2 mutants across a titration.** Representative western blot analysis of different overexpressed TMPRSS2 variants used in Figure 3D.


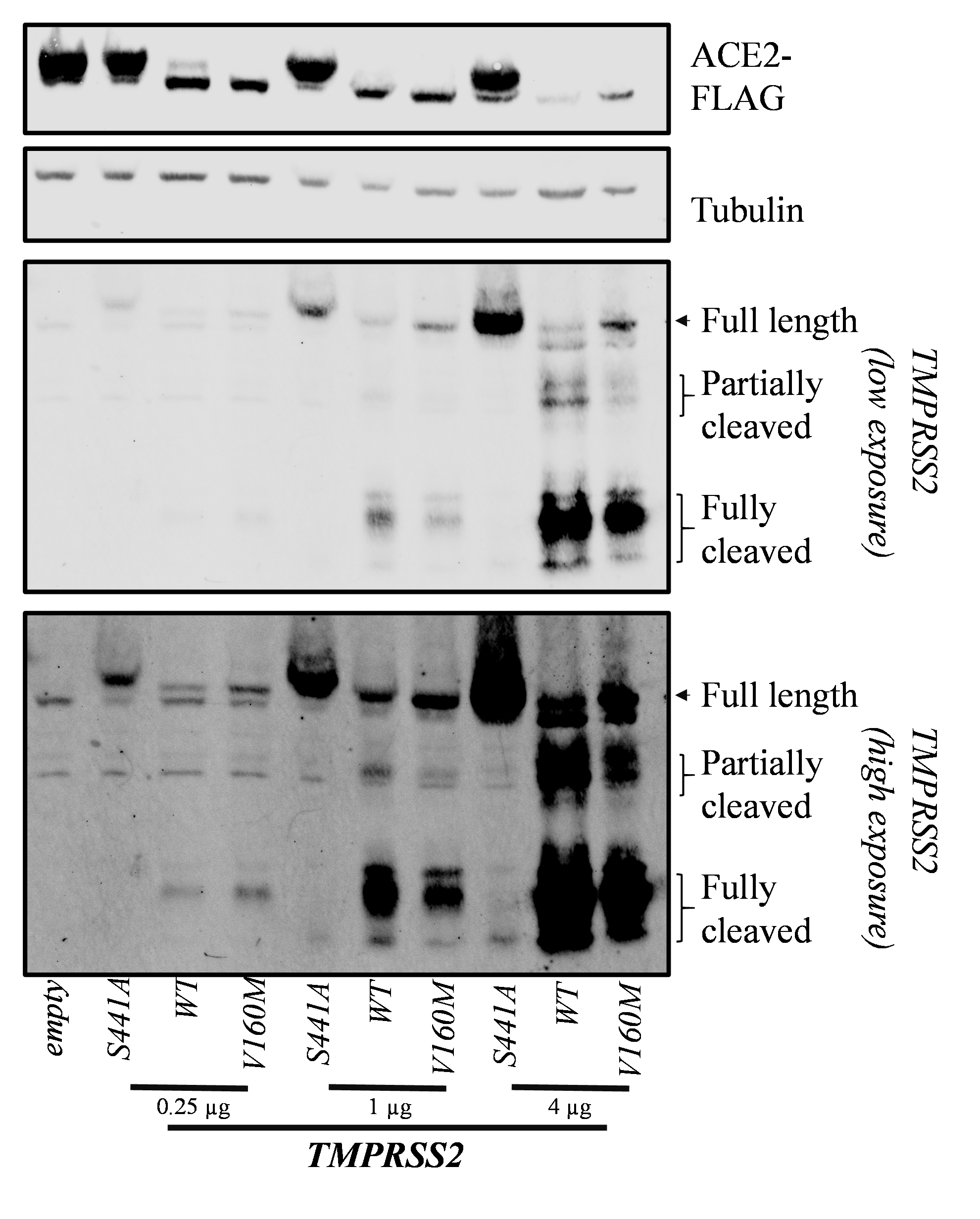


**Supplementary Figure S5. TMPRSS2 autocleavage phenotype in cells endogenously expressing TMPRSS2.** Comparative TMPRSS2 cleavage in Calu-3 and Caco-2 cells compared to overexpressed TMPRSS2 in HEK 293Ts. µg values indicate amounts of TMPRSS2 plasmid transfected.


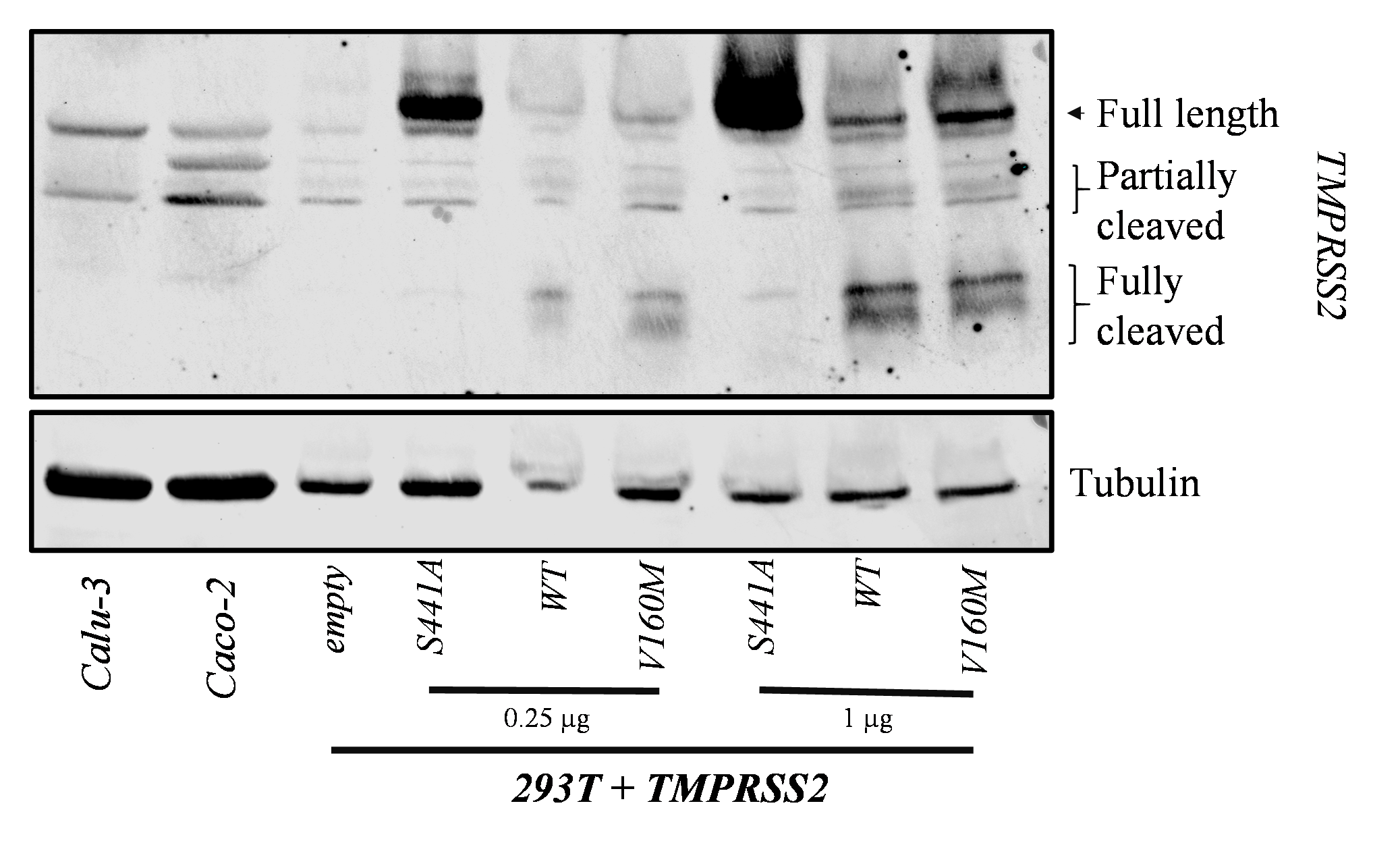

Supplement: Supplementary file 1 [file mmc1.docx]
